# A study of screw placement to obtain the optimal pull-out resistance of lumbar pedicle screws—analysis of Hounsfield units measurements based on computed tomography

**DOI:** 10.1186/s12891-022-05074-6

**Published:** 2022-02-07

**Authors:** Dachuan Li, Chi Sun, Jianyuan Jiang, Feizhou Lu, Xinlei Xia, Hongli Wang, Fei Zou, Xiaosheng Ma

**Affiliations:** Department of Orthopedics, Huashan Hospital, Fudan University, No.12, middle Urumqi Road, Jing’an District, Shanghai, 200040 China

**Keywords:** Hounsfield units, Bone mineral density, Posterior lumbar interbody fusion, Pedicle Screws

## Abstract

**Objective:**

The screw path of lumbar pedicle screws in the vertebral body has certain variability. It is not clear whether the screw paths in different directions can obtain the same pull-out resistance. This study intends to use CT (Computed Tomography) to measure the Hounsfield unit (HU value) around the screw paths in different parts of the lumbar vertebral body to obtain the bone mineral density value of the corresponding parts which will provide some reference for the direction of lumbar pedicle screw placement.

**Methods:**

This retrospective study included 200 patients with lumbar degenerative diseases selected randomly from the case base and the patient’s basic information was recorded. L1-L5 vertebral body was divided equally into the upper, middle and lower 1/3, which was consistent with the three sagittal entry directions of the pedicle screw head tilt, parallel endplate and caudal tilt, and the HU values were measured by CT cross-sectional scanning to indirectly reflect the local bone density values. The paired t-test (randomized block experiment) was used to compare the HU values of the upper, middle and lower 1 / 3 parts, with *P* < 0.05 being considered statistically significant.

**Results:**

Comparison of HU values in different parts of each vertebral body revealed that HU values in the middle 1/3 of the L1,L2 (163.88 ± 58.44 and 152.94 ± 59.45) and in the lower 1/3 of the L4 (149.86 ± 60.18) were higher than in the other two parts of the vertebral body of the same segment(*P* < 0.0001,*P* = 0.0069 and *P* = 0.0024, respectively); According to the results of each stratification, patients with younger age and better bone condition had higher HU values in the middle 1/3 of L1 and L2, and higher HU values in the lower 1/3 of L3, L4 and L5; With the increase of age, the decrease of bone condition and the difference of HU value in each vertebral body gradually decreased.

**Conclusion:**

Although further follow-up studies are needed, based on the analysis of the statistical results, we speculate that from the perspective of obtaining the best pull-out resistance of the lumbar pedicle screws, the placement direction of L1 and L2 in the sagittal position may be as parallel to the endplate as possible; L3, L4, and L5 may be as appropriate as possible to the tail tilt theoretically.

## Introduction

Posterior lumbar interbody fusion (PLIF) is a commonly used surgical method for spinal surgery to treat degenerative diseases of the lumbar spine, which can achieve better prognosis in patients [1, 2].Since the first operation was performed in 1940, the majority of cases worldwide have been performed using this method. Statistics showed that the number of lumbar fusion operations worldwide doubled between 1996 and 2002 [3]. However, postoperative screw loosening is the most common complication of this surgery, especially in patients with osteoporosis and poor bone quality, which is the important reason that makes spinal surgery fixation fail. In serious cases, a further revision surgery is often required. Clinical studies have found that the rate of screw loosening in normal patients is about 1–27%, while the rate of screw loosening in patients with osteoporosis is as high as 60% [4–6]. With the aging of the population, osteoporosis has now become one of the most common diseases in the elderly, affecting 200 million people on a global scale. Therefore, in these patients undergoing posterior lumbar fusion surgery, it is necessary to select the appropriate method to avoid screw loosening as much as possible, and to enhance the screw pull-out resistance.

There are two most important factors affecting the strength of the pedicle screw (resistance to pullout): the quality of the vertebral bone (bone mineral density, BMD) and the trajectory of the screw [7]. At present, it has been confirmed that the BMD of the vertebral body is positively correlated with the pullout force of the screw [8]. Many studies in recent years have shown that the Hounsfield units (HU) value of the vertebral body measured by CT scans could also help detect BMD. Moreover, a large number of analyses have confirmed that the HU value of the vertebral body was positively correlated with BMD and had a certain conversion relationship [9]. In addition, CT not only has no measurement limitations, but it can also evaluate the bone mineral density in a specific area, and some studies have used this feature to measure the HU value of screw trajectory [10], which found that HU value was also positively correlated with the anti-pull-out strength of the screw and could be used as an indicator for predicting pedicle screw loosening [11, 12].

In the current posterior lumbar spine surgery, the junction between the midline of the transverse process of the posterior middle section of the vertebral body and the outside of the facet joint is usually selected as the screw insertion point [13], but the insertion direction of the screw has a certain degree of variability. With the insertion of the screw, theoretically the head of the screw can be tilted in the direction in front of the vertebral body, so as to obtain a better fixation angle. However, no studies have been reported to use CT to analyze the HU values around the screw path in different directions in front of the vertebral body to select the direction of screw placement with the best pull-out resistance. Therefore, it is not clear whether the screw paths in different directions can obtain the same pull-out resistance. As a result, preoperative application of this type of assessment will help improve the stability of pedicle screw placement in vivo and reduce the risk of screw loosening.

The purpose of this study is to measure the Hounsfield units (HU value) around the screw path of different parts in front of each lumbar vertebra in patients with lumbar degenerative diseases, so as to obtain the best direction of screw placement to obtain the best pull-out resistance, and to provide some reference for the direction of lumbar pedicle screw placement.

## Materials and methods

This study was a retrospective cohort analysis of the data collected, approved by the appropriate ethics committee of our hospital and performed in accordance with the ethical standards laid down in an appropriate version of the 1964 Declaration of Helsinki.

### Patients

We selected 200 patients randomly from the case base which contains the 535 patients that were admitted to the hospital for posterior lumbar decompression and fusion for lumbar degenerative diseases from January to December 2017.

### Inclusion criteria

1. lumbar degenerative diseases, including degenerative lumbar spinal stenosis, degenerative lumbar spondylolisthesis, lumbar disc herniation, etc., and posterior lumbar fusion pedicle screw fixation
2. No previous history of spinal surgery
3. Complete preoperative CT scan data of lumbar spine;

### Exclusion criteria

1. Patients with spinal fractures, a history of spinal surgery, infections, tumors, deformities, ankylosing spondylitis, and significant osteogeny of the facet joints
2. Patients with incomplete CT scan data of lumbar spine

A total of 200 patients were included (103 men and 97 women; Age: 52.57 ± 16.10 years; Range, 15–85 years), patients’ age, gender, disease diagnosis, osteoporosis and other basic information were recorded and stratified for comparison.

### Evaluation method of Regional HU

**Fig. 1:**
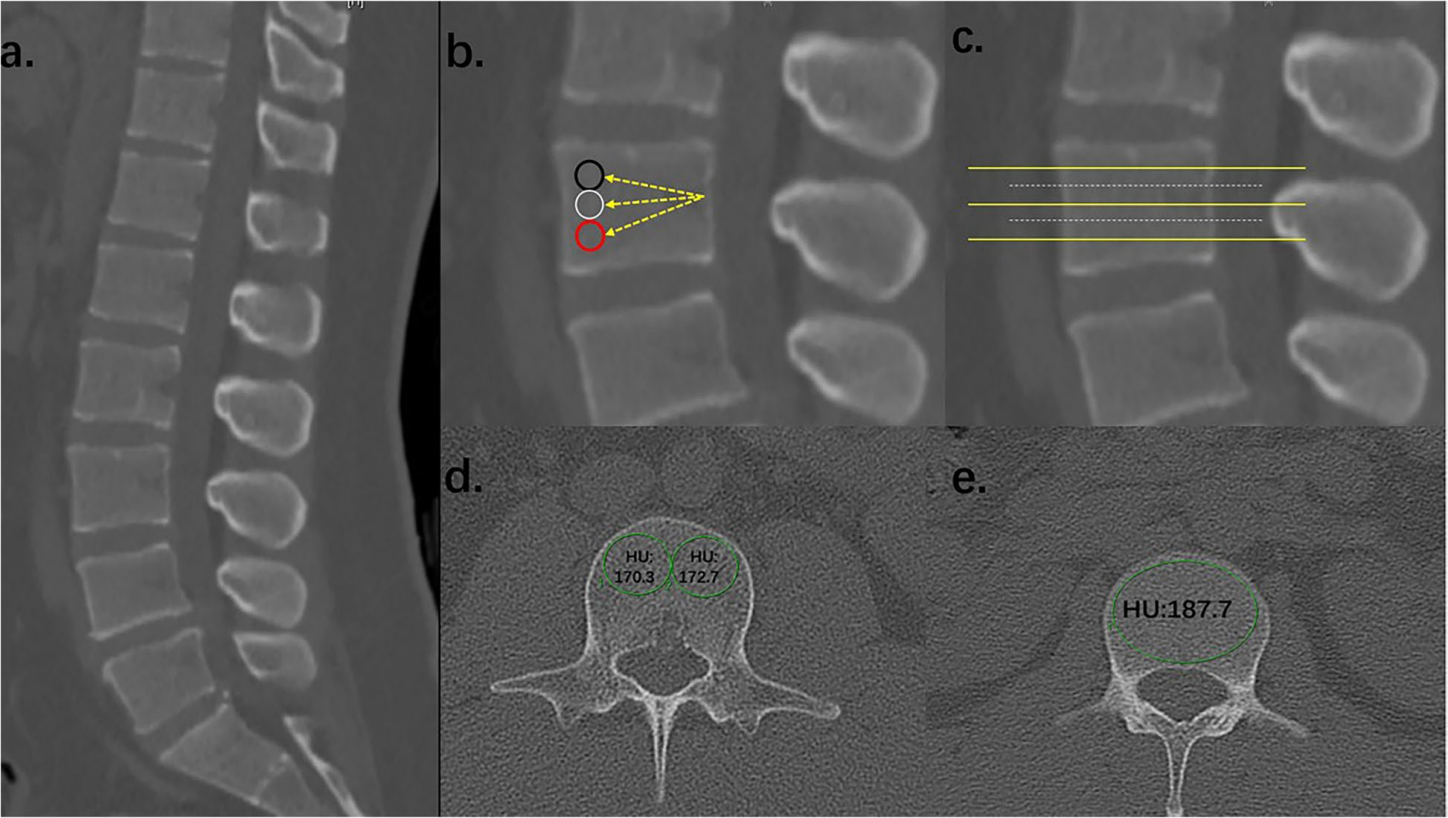
Technique of obtaining HU values is demonstrated (**a**-**d**). **b** The three arrow directions indicated the head tilt, parallel endplate and tail tilt in the direction of screw placement, and the circle indicated the area where the screw head was located. **c** At the level of the upper, middle and lower body, the vertebral body (L3) was equally divided into three parts (white dotted line) in the craniocaudal direction. A median line was taken from each of the three sections of the sagittal image to select the transects measured (yellow line). **d** Two elliptical regions of interest (ROI) were placed on the vertebral cross-sectional images on the left and right sides, and the software automatically calculated the mean HU value of the ROI. The vertebra’s bone density was measured in the cancellous bone surrounded by an oval contour (green oval) excluding cortical bone and posterior venous plexus at the level of the middle body. **e** Example of the measurement of HU value to help assess osteoporosis: In a cross-sectional image at the level of the midbody, the mean HU value of L1 ROI was 187.7

CT images of L1-L5 were collected from all patients included in the study on the Picture Archiving and Communication Systems (PACS, GE Healthcare, IL, USA), and each vertebral body was equally divided into upper, middle and lower 1/3, which was consistent with the sagittal entry directions of pedicle screw head tilt, parallel endplate and caudal tilt. The HU values in the upper, middle, and lower 1/3 of the L1-L5 vertebral body and in the left and right regions of the anterior 1/2 of the vertebral body were measured by CT transverse scanning. The specific measurement method referred to the previous research and was modified [14]. Firstly, the upper, middle, and lower 1/3 of the vertebral body were located in the sagittal view, and in the transverse CT image of the located vertebral body, an elliptical area of interest (ROI) was placed on each side of the anterior 1/2 of the vertebral body to represent the fixation area of the screw head after screw placement (Fig. 1). The ROI included cancellous bone trabecular bone, but did not include cortical bone and heterogeneous areas, such as posterior venous plexus, bone island, compressed bone, etc. The HU value of the ROI selected in the CT transverse image was automatically calculated by the system. Zhang et.al [15] found that there was no difference in the HU values of the left and right sides of the same vertebral body in the same cross-section. Therefore, we averaged the HU values of the left and right ROI of the positioned cross-section respectively to represent the average HU value at 1/2 of the anterior part of the three parts of the upper, middle and lower parts of the vertebral body, thereby reflecting the bone mineral density of the vertebral body in this area indirectly [16]. The measurement was performed by two spine surgeons familiar with the anatomy of the lumbar spine and screw placement surgery. Each parameter in each vertebra was measured twice, and the average of the two times was used as the final value.

### Assessment of osteoporosis

According to the measurement and diagnosis of bone mineral density by lumbar QCT (Quantitative Computed Tomography), the thresholds of osteoporotic vertebrae were divided into normal (BMD > 120 mg/cm^3^), osteopenia (low bone mass) (80 mg/cm^3^ ≤ BMD ≤ 120 mg/cm^3^) and osteoporosis (BMD < 80 mg/cm^3^) [17]. Generally, L1 and L2 are selected to measure the BMD of these two vertebral bodies respectively, and the average value is taken as the basis for diagnosis [18]. In our study, we measured the HU values of L1 and L2 in the middle section of the two vertebral bodies (Fig. 1e) and took the mean values of both, representing the average HU values of the entire lumbar spine, and converted them into BMD values represented by QCT according to the conversion formula obtained from the previous study on the correlation between HU values of CT and QCT bone mineral density [19]. Combined with the diagnostic criteria, the patients were approximately divided into normal bone, low bone mass and osteoporosis groups.

### Statistical Analysis

The continuous variable results in descriptive statistics were expressed as mean ± standard deviation. The Shapiro-Wilk test was used to verify the normal distribution of continuous variables. Statistical analysis was performed using SPSS software (version 26, USA). Randomized block test (two-way ANOVA) was used to compare the HU values in the anterior 1/2 of the upper, middle, and lower 1/3 of the L1-L5 vertebral bodies of different patients, and the Tukey test was used to compare the HU values in these three parts pairwise. *P* < 0.05 was considered a statistically significant difference.

## Results

**Fig. 2:**
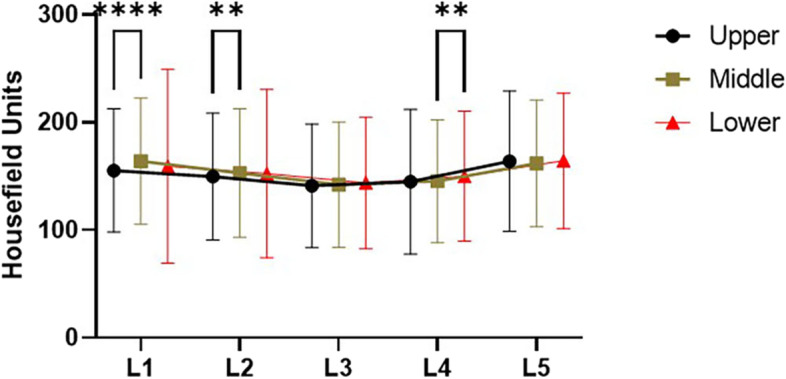
The variation trend of HU values in different parts of L1-L5 vertebral body in all patients

A total of 200 patients were eventually included, and the characteristics of all patients were summarized in Table 1. The overall mean age was 52.57 ± 16.10, and the average HU value was 162.30 ± 56.76. The variation trend of HU values in different parts of L1-L5 vertebral body and the comparison results showed that the mean HU value of the middle 1/3 of L1 and L2 was significantly higher than the upper 1/3 (*P* < 0.0001 and *P* = 0.0069, respectively), and the average HU value of the lower 1/3 of L4 was considerably higher than the middle 1/3 of L4 (*P* = 0.0024) and there was no significant difference in the HU values of other vertebral bodies (L3: *P* = 0.0790, L5: *P* = 0.3540).(The results of multiple comparisons were shown in Fig. 2).

**Table 1:** Patient demographics

Basic case information	Mean age	Number	Mean HU value
Gender			
Male	**50.12 ± 17.85**	**103**	**167.74 ± 54.67**
Female	**55.21 ± 13.46**	**97**	**156.47 ± 58.66**
Age			
< 40	**29.02 ± 6.39**	**43**	**221.23 ± 40.06**
40 ~ 50	**44.89 ± 2.86**	**37**	**192.09 ± 42.64**
50 ~ 60	**54.78 ± 2.72**	**45**	**145.38 ± 39.30**
60 ~ 70	**64.40 ± 2.91**	**47**	**131.47 ± 39.38**
≥ 70	**76.33 ± 4.44**	**28**	**120.59 ± 45.16**
Osteoporosis			
Normal	**43.25 ± 15.14**	**99**	**207.89 ± 39.25**
Osteopenia	**59.24 ± 10.67**	**71**	**132.39 ± 14.06**
Osteoporosis	**68.07 ± 8.08**	**30**	**79.92 ± 15.92**
Disease types			
Non-lumbar spondylolisthesis	**63.14 ± 10.53**	**152**	**133.77 ± 42.87**
Lumbar spondylolisthesis	**51.67 ± 3.44**	**48**	**181.62 ± 40.83**
Lumbar spondylolisthesis and osteoporosis	**64.73 ± 6.21**	**16**	**86.45 ± 13.76**
Total	**52.57 ± 16.10**	**200**	**162.30 ± 56.76**

### According to the stratification of variables

#### Gender

**Fig. 3:**
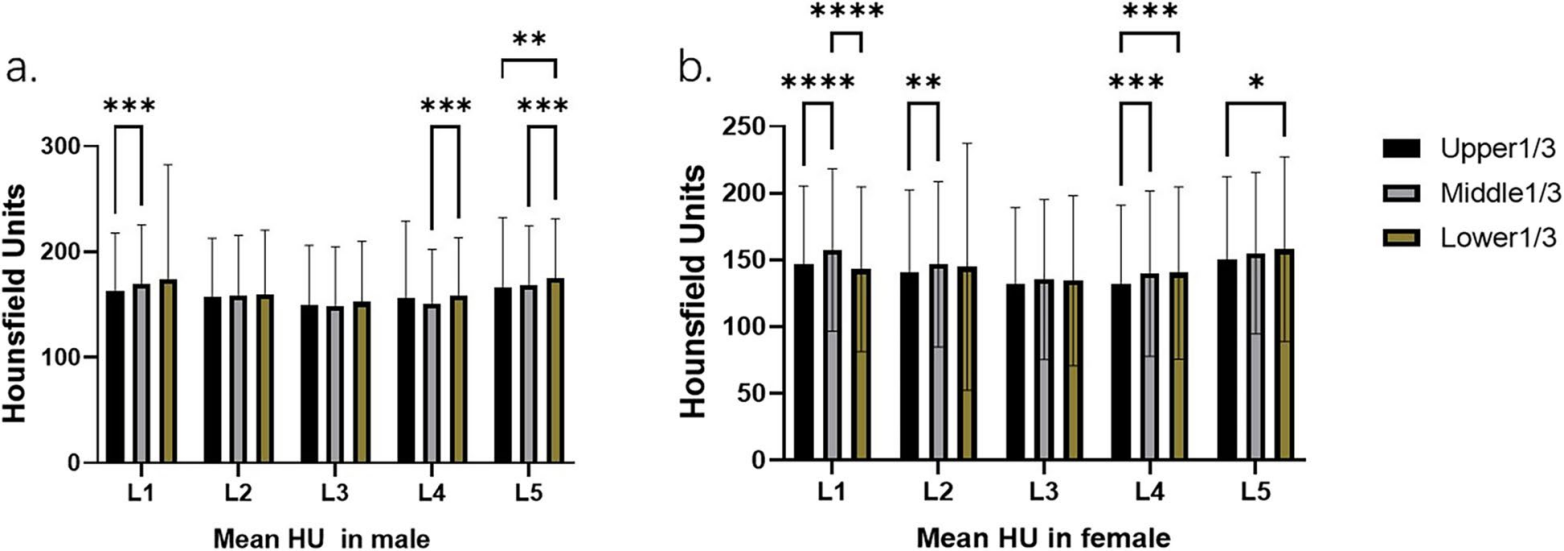
Comparison of HU values of L1-L5 parts in male (**a**) and female (**b**)

In 103 male patients and 97 female patients, we found that the mean HU values in the middle 1/3 of L1 and the lower 1/3 of L4 and L5 were significantly higher than the rest of the vertebral body of the same segment in both men and women (Male: L1: *P* < 0.0001, L4: *P* = 0.0156, L5: *P* = 0.0050; Female: L1: *P* < 0.0001, L4: *P* < 0.0001, L5: *P* = 0.0142). In addition, in female patients, the mean HU value of the middle 1/3 of L2 was dramatically higher than that of the upper 1/3 (*P* = 0.0011), and the HU value of the middle 1/3 of L4 was also higher than that of the upper 1/3 and had no noticeable difference with that of the lower 1/3(*P* = 0.0002). There was no statistical difference in the HU values of the other vertebral parts (Male: L3: *P* = 0.0732, Female: L3: *P* = 0.6368). (The results of multiple comparisons were shown in Fig. 3).

#### Age

**Fig. 4:**
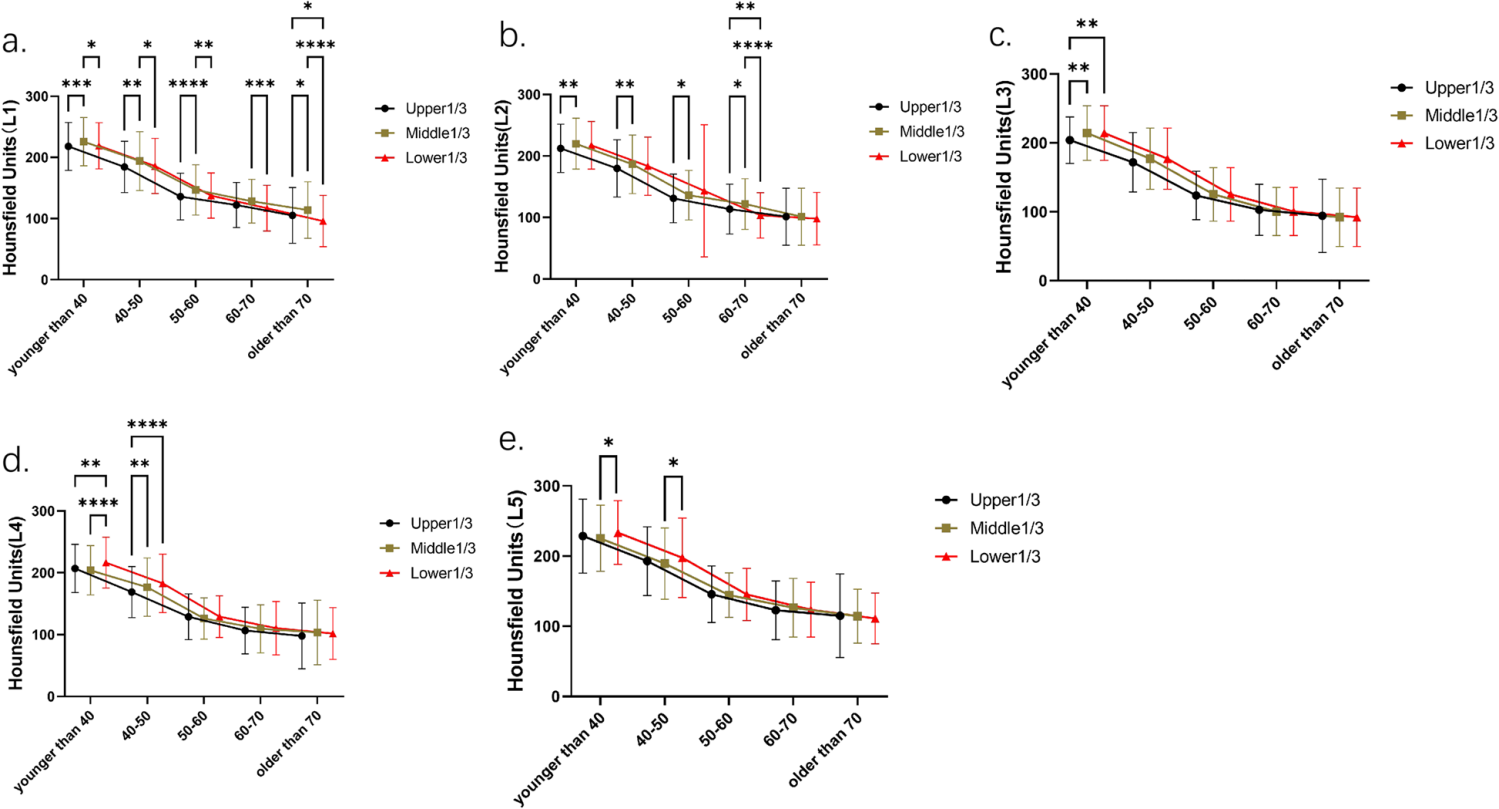
Comparison and trend of HU values in each part of L1-L5vertebral body in patients of different ages (**a**-**e**). As shown in the figure, the HU values of the middle 1/3 of L1, L2 and the lower 1/3 of L3-L5 were higher; with the increase of age, the HU values gradually decreased, accompanied by the difference of the HU values in each part of the same vertebral body, which L3-L5 was the first to show

According to the age range of patients (15–85 years old), the patients were divided into five age groups: less than 40 years old, 40 to 50 years old, 50 to 60 years old, 60 to 70 years old, and more than or equal to 70 years old. The results showed that patients younger than 50 years old had significant differences in various parts of the vertebral body. The middle 1/3HU values of L1 and L2 and lower 1/3HU values of L4 and L5 were dramatically higher than those of other parts of the same segment (younger than 40: L1, L2, L4, L5: *P* = 0.0050, *P* < 0.0001, *P* < 0.0001, *P* = 0.0048, respectively; 40–50 years: L1, L2, L4, L5: *P* = 0.0020, *P* = 0.0177, *P* < 0.0001, *P* = 0.0119, respectively) (Fig. 4a,b,d,e). The HU in the middle 1/3 of L3 was also significantly higher in patients younger than 40 years (*P* < 0.0001). With the increase of age, there were only differences in L1 and L2 among patients aged 50–70 years, and the mean HU value of the middle 1/3 was significantly higher (50–60 years: L1: *P* < 0.0001, L2: *P* = 0.0063; 60–70 years: L1: *P* = 0.0010, L2: *P* = 0.0075) (Fig. 4a,b) among the elderly patients who were 70 years old or older, only the middle 1/3 of L1 had a statistically higher mean HU than the rest of the same vertebral segment (*P* < 0.0001). There was no statistical difference in the HU values of the other vertebral parts (L2-L5: *P* = 0.8240, *P* = 0.3124, *P* = 0.3376, *P* = 0.8281, respectively). (The results of multiple comparisons were shown in Fig. 4).

### Osteoporosis

**Fig. 5:**
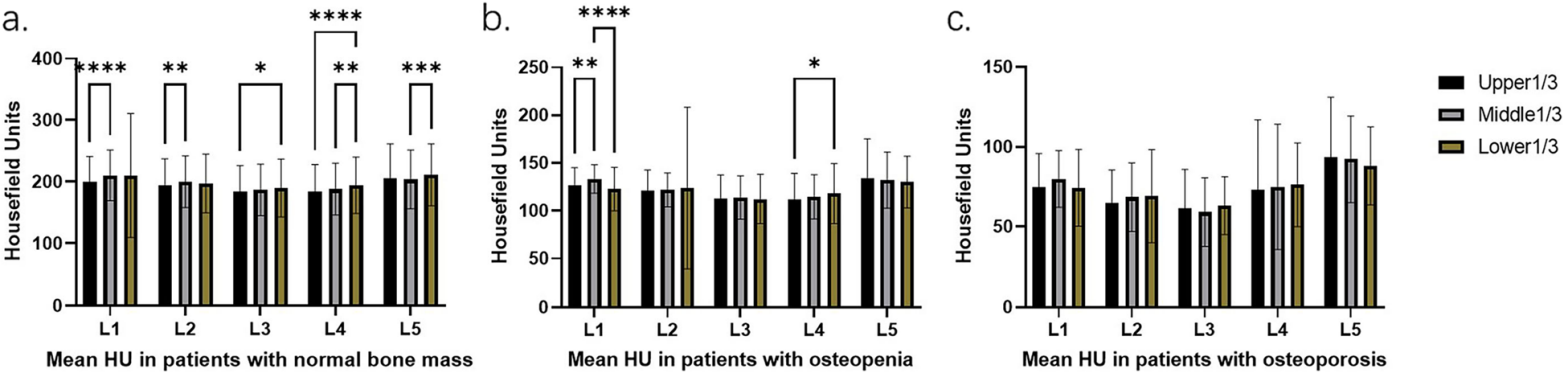
Comparison of HU values of L1-L5 parts in different bone conditions groups (**a**-**c**)

In the grouping based on patients’ bone condition, 99 patients with normal bone had significant differences in each part of the lumbar spine. The mean HU values in the middle 1/3 of L1 and L2 and the lower 1/3 of L3, L4 and L5 were dramatically higher than those in other parts of the same segment (L1-L5: *P* < 0.0001, *P* = 0.0312, *P* = 0.0113, *P* < 0.0001, *P* = 0.0064, respectively) (Fig. 5a), while 71 patients with osteopenia showed noticeable differences in mean HU values only between the middle 1/3 of L1 and the lower 1/3 of L4 (L1: *P* < 0.0001, L4: *P* = 0.0078) (Fig. 5b), there were no significant differences in lumbar vertebra segments among the 30 patients with osteoporosis (L1-L5: *P* = 0.1592, *P* = 0.5783, *P* = 0.2712, *P* = 0.8436, *P* = 0.5345, respectively) (Fig. 5c).

### Diseases types

**Fig. 6:**
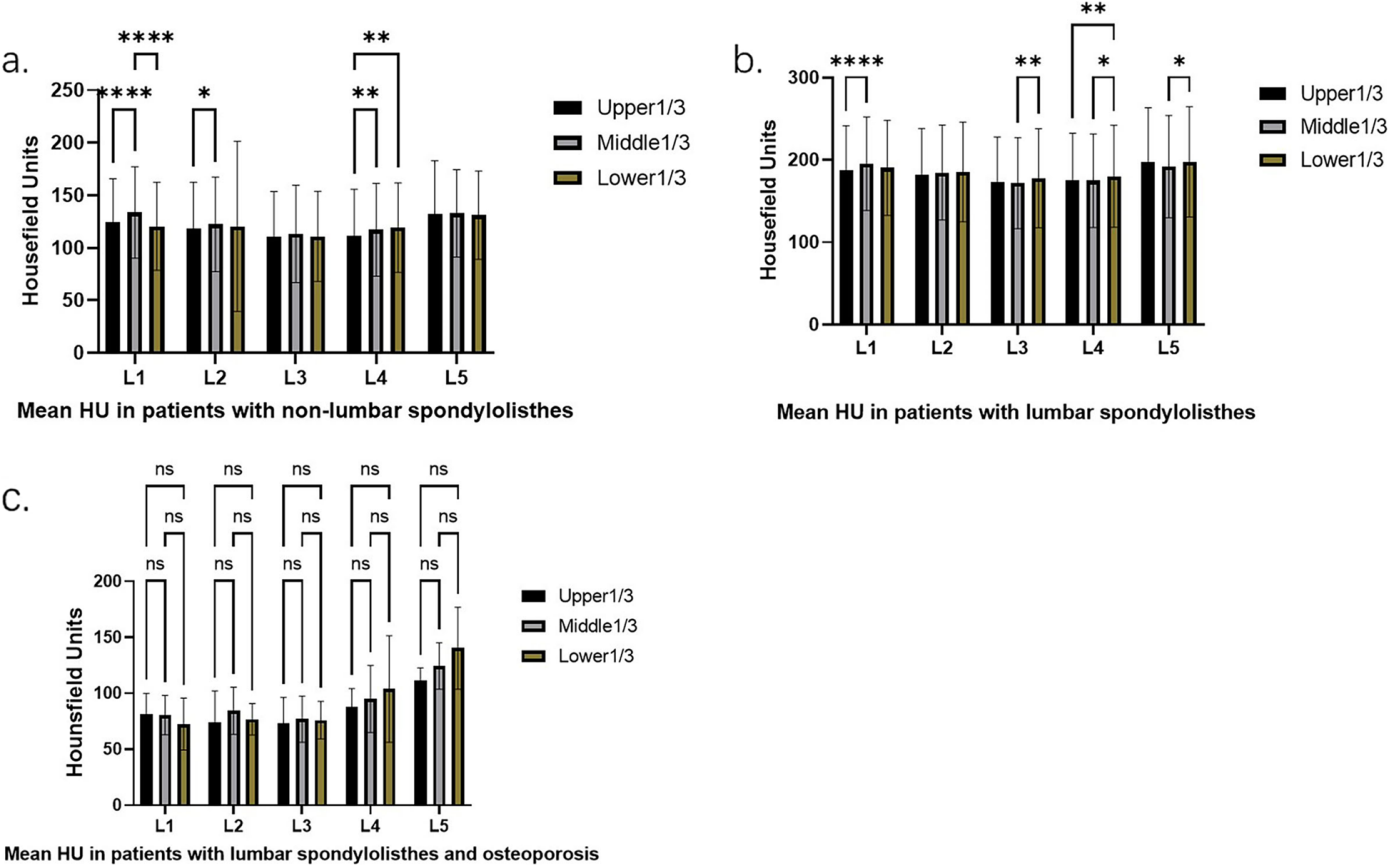
Comparison of HU values of L1-L5 parts in different disease types groups (**a**, **b**). There was no difference in different parts of L1-L5 for patients with lumbar spondylolisthesis and osteoporosis (**c**)

The results showed that in 152 non-lumbar spondylolisthesis patients and 48 patients with lumbar spondylolisthesis, the HU value of the middle 1/3 of L1 and the HU value of the lower 1/3 of L4 were dramatically higher than the other parts of the same segment (non-lumbar spondylolisthesis: L1: *P* < 0.0001, L4: *P* = 0.0011; lumbar spondylolisthesis: L1: *P* = 0.0012, L4: *P* = 0.0041). In addition, patients with non-lumbar spondylolisthesis had significantly higher HU values in the middle 1/3 of L2 (*P* = 0.0359), and patients with lumbar spondylolisthesis had higher HU values in the lower 1/3 of L3 and L5, and both had significant differences (*P* = 0.0050 and *P* = 0.0081, respectively). There were no noticeable differences among the other parts of the vertebral body (non-lumbar spondylolisthesis: L3: *P* = 0.2231, L5: *P* = 0.8037; lumbar spondylolisthesis: L2: *P* = 0.0632). (The results of multiple comparisons were shown in Fig. 6).

## Discussion

Traditional lumbar pedicle screw fixation has become a common surgical procedure for the treatment of lumbar degenerative diseases, and one of the most important factors affecting pedicle screw strength (pull-out resistance) is screw placement trajectory [7]. Most of the screw path are located in the vertebral cancellous bone. Screw loosening is highly common as BMD decreases, which weakens the fixation effect [8]. Therefore, it is necessary to determine the bone density of the screw placement area before the operation in order to select the screw placement track with better bone condition [20]. At present, the position of the insertion point of the PLIF is acknowledged, located at the junction of the midline of the transverse process in the posterior midsection of the vertebral body and the outside of the facet joint [13]. This allows the trajectory of the screw at the pedicle and the posterior aspect of the vertebral body almost the same after being inserted into the vertebra. Although the direction of screw placement for most surgeries is parallel to the endplate, due to the influence of many factors, such as the curvature of the patient’s lumbar spine, the model of the implanted fusion cage, etc., the trajectory direction of the screw head at the anterior aspect of the vertebral body has a certain degree of variability [21]. At the same time, it is not clear whether screw trajectories in different directions can obtain the same pull-out resistance. We think that it is necessary to analyze the HU values (indirect BMD results) in the anterior region of the screw inserted at the anterior aspect of the vertebral body. Therefore, this study is the first to measure the HU values around the screw path of the lumbar pedicle screws in different directions at the anterior aspect of the vertebral body and analyzes the optimal screw placement direction to obtain the optimal pull-out resistance.

According to the overall results of all patients in this study, the parts with higher HU values were located in the middle 1/3 of L1 and L2, the lower 1/3 of L4, and the changing trend of HU values in different parts of L1-L5 vertebral body was higher in the middle 1/3 of upper lumbar vertebra and higher in the lower 1/3 of lower lumbar vertebra. The HU values of different segments from L1 to L5 were the lowest in L3 and the highest in L5, which was consistent with the results of previous studies on the mean HU values of each vertebral body [14]. It was considered that the middle 1/3 of L1 was the most favorable for screw placement, with the direction parallel to the endplate. It was also found in the study that the younger the patients or the better the BMD, the more significant the difference of HU values in different parts of the vertebral body was. The HU values in the middle 1/3 of L1 and L2 was higher, while the HU values in the lower 1/3 of L3-L5 was higher. Combined with these results, we hypothesized that the transition of high HU from the middle of the upper lumbar spine down to the lower lumbar spine might be due to physiological and biomechanical reasons. These young patients with better bones did not have more serious structural changes in the lumbar spine. Therefore, their high HU values of L1 and L2 were concentrated in the middle of the vertebral body to maintain the stability of the entire lumbar spine in a more balanced way, while the lower lumbar spine, especially the lower part of the lower lumbar spine, carried a large portion of the body weight to support normal and weight-bearing activities, so the HU value was relatively increased [22]. At the same time, we observed a deterioration in bone mass and a decrease in the mean HU value of each vertebral body with increasing age, which was consistent with the findings of Schreiber JJ [9]. In addition, it was also found in our study that the differences of HU values in different parts of the vertebral body also decreased, and this change first appeared in the lower lumbar (L4, L5), and involving the upper lumbar gradually, finally only osteoporosis group without the high HU value in the middle of L1 results, showing the whole lumbar parts HU values were no difference. We considered that such a result might be due to the fact that the older the person was, the more serious the degeneration of bone was, and the more likely and serious the degenerative changes of the structure of the lumbar spine were to occur, while L4 and L5 were the more prone and easily affected first [23]. As a result, the degeneration of the lower lumbar spine resulting in the decrease of the HU value difference in various parts of the vertebral body was earlier and more obvious, so that the degeneration of the entire vertebral body to a low HU value was the first manifestation. From the perspective of different genders and different diseases, the results were almost consistent with the overall performance. The pronounced reasons for the subtle differences remain unclear and require further investigation. We analyzed that this difference might be related to other factors, such as different lifestyle, physical activity, and different changes in the vertebral body or endplate. The analysis reason might be that the difference was not obvious due to the small number of cases.

Previous related studies have suggested that the value of BMD around the screw track can affect or even predict the loosening of the screw [24]. DEXA is the current gold standard for BMD measurement and osteoporosis screening. However, the accuracy of BMD measurement by DEXA is affected by degenerative changes of the lumbar spine, scoliosis, osteogeny, compression fracture, calcification and other aspects, and cannot distinguish between cortical bone and cancellous bone. Some studies have found that the detection of BMD based on the HU value measured not only had a certain linear relationship with the BMD evaluated by DEXA and QCT, but also could be used for auxiliary diagnosis of osteoporosis, while avoiding the above shortcomings [9]. It is noteworthy that many new reports about the clinical application of HU were emerged in recent years. Matsukawa et.al [11] found that the pull-out resistance of the screw was correlated with the HU by measuring the screw path HU and the insertion torque of the inserted screw. With a low HU value, the pull-out resistance of the screw is also low, indicating the risk of screw loosening. Many other similar studies have also concluded that preoperative CT measurements of HU could predict screw loosening [25, 26]. Zhang.et.al [27] compared the HU values of the entire screw trajectory between the cortical screw and the traditional screw, concluded that the pull-out resistance of the cortical screw was stronger. Wichmann et.al [28] found that the bone mineral density at the pedicle was more strongly correlated with the pull-out resistance of the inserted screws compared with that inside of the vertebral body. Meanwhile, it was also found that the bone mineral density of the pedicle screws in the vertebral body segment (the area of the screw head) was significantly different from that at the pedicle, which was lower than that at the vertebral pedicle. On this basis, Fei Xu et.al [29] respectively analyzed the relationship between the HU value at the pedicle and inside the vertebral body and screw loosening, and also found that the HU value at the pedicle was more correlated with screw loosening than the HU value in the vertebral body, and the prediction was more sensitive. However, it was also found that the HU value in the vertebral body had the same predictive value. The results of the research of these scholars have confirmed that the value of HU (around the vertebral body or screw path) related to the loosening and pull-out resistance of the screw. But to date, no study has been conducted on the basis of these findings to select a better and more suitable screw placement direction. Therefore, we believe that the analysis of the screw trajectory in different directions, especially the screw head area in the anterior part of the vertebral body, may provide new findings, which can provide some reference for the selection of a suitable screw placement direction.

This study has the following deficiencies. Firstly, this study is a retrospective study, and more prospective studies are needed to verify the relationship between HU values in different directions of the vertebral body and pull-out resistance after screw placement. At the same time, these patients need to be further followed up clinical and radiological follow-up data to assess the screw placement status. Secondly, we measured the HU value by manually selecting ROI. Although in this study, multiple measurements and averaging were used to improve inter-observer and intra-observer reliability, this might cause concerns about the repeatability of these results. In addition, we did not conduct a formal BMD measurement for the patients, but relied on the HU conversion formula to convert into QCT values, rather than the bone density values measured by DEXA as the diagnostic criteria, so as to determine patients with different bone conditions, which might lead to diagnostic errors. Finally, we measured the HU values in three of the cross-sections after trisection of the vertebral body, which represented regional HU values, reflected the local two-dimensional bone mineral density, in the actual operation of screw placement is three dimensional, so by only a few cross-sectional data might obscure or ignore other potential factors that affect the HU.

**Fig. 7:**
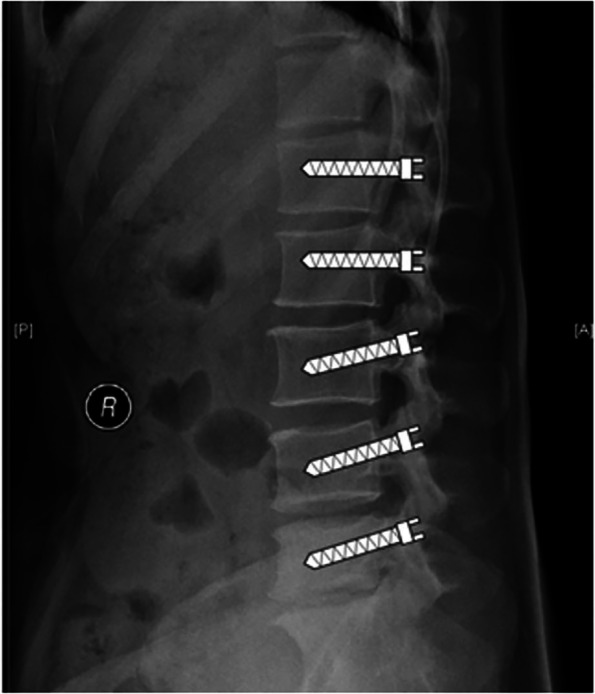
Based on the analysis of statistical results, the optimal placement direction of the pedicle screws in each vertebral body of the lumbar spine and the direction in patients with lumbar spondylolisthesis and osteoporosis is consistent with the criteria concluded

Based on the above analysis, we can consider to improve the direction of screw placement inside the vertebral body preoperatively. According to our statistical results, the sagittal placement direction of pedicle screws in patients with L1 and L2 lesions can be parallel to the endplate, and the patients with L3, L4 and L5 lesions can be appropriately caudally tilted (Fig. 7). Additionally, we conducted a separate stratified analysis for patients with lumbar spondylolisthesis and osteoporosis. Osteoporosis is one of the important risk factors for osteoporotic vertebral compression fractures [30], and VCF (vertebral compression fracture) is also a common postoperative complication of PLIF in spine surgery [31]. Besides, previous studies have confirmed that lumbar spondylolisthesis is an independent risk factor for osteoporotic vertebral compression fractures, and lumbar spondylolisthesis is not only associated with disc degeneration but also with curvature of the spine, such as lumbar lordosis or chest kyphosis increase, which could affect sagittal curvature or spinal sagittal balance and cause VCF [32]. These patients need to use internal fixation surgery for reduction to improve symptoms and function, and obtain stability, so a higher requirement for screw pull-out resistance is often needed to further avoid surgical failure. Although there was almost no difference in different parts of the vertebral body for elderly patients, osteoporosis, and patients with lumbar spondylolisthesis and osteoporosis (Fig. 6c), the results were found to be almost identical, which may also be related to the small number of samples by analyzing the variation trend of HU values in these patients and the overall sample, and the standard we concluded could still be used for evaluation. Therefore, cortical bone screws, injectable fillers at the surgical site or adjacent vertebral segments and adjusting the size and shape of screws should be considered for aged patients and osteoporotic patients according to the actual situation of the operation to ensure a better pull-out resistance of the screws. Simultaneously, special attention should be paid to the patients with lumbar spondylolisthesis and osteoporosis to prevent vertebral fracture and other postoperative complications.

## Conclusion

In general, this study illustrated the difference of HU values in different parts of the anterior vertebral body from L1 to L5 for the first time and provided certain reference for the direction of lumbar pedicle screw placement. Although further follow-up studies are needed, based on the analysis of the statistical results, we speculate that from the perspective of obtaining the best pull-out resistance of the lumbar pedicle screws theoretically, the sagittal placement direction of pedicle screws for L1 and L2 vertebral bodies may be parallel to the endplate as far as possible, and the sagittal placement direction of pedicle screws for L3-L5 vertebral bodies may be caudally inclined; For patients with low HU values such as advanced age and osteoporosis, there was no significant difference in the statistical results of each part, but the best placement direction may still be selected by referring to this result and combining with the actual situation of the operation. At the same time, additional measures should be taken to enhance the pull-out resistance of the screw to avoid screw loosening.

## Data Availability

The datasets used and analyzed during the current study are available from the corresponding author on reasonable request.

## Abbreviations

CT: Computed Tomography
HU: Hounsfield Units
PLIF: Posterior lumbar interbody fusion
BMD: Bone mineral density
DEXA: Dual-energy X-ray absorptiometry
WHO: World Health Organization
QCT: Quantitative Computed Tomography
VCF: Vertebral compression fracture
ROI: Regions of interest
